# How Do Scientists Define Openness? Exploring the Relationship Between Open Science Policies and Research Practice

**DOI:** 10.1177/0270467616668760

**Published:** 2016-06-01

**Authors:** Nadine Levin, Sabina Leonelli, Dagmara Weckowska, David Castle, John Dupré

**Affiliations:** 1 University of California Los Angeles, CA, USA; 2 University of Exeter, Exeter, UK; 3 University of Sussex, Brighton, UK; 4 University of Victoria, Victoria, British Columbia, Canada

**Keywords:** open science, open access, life sciences, research practice, science policy, open data, research infrastructure

## Abstract

This article documents how biomedical researchers in the United Kingdom understand and enact the idea of “openness.” This is of particular interest to researchers and science policy worldwide in view of the recent adoption of pioneering policies on Open Science and Open Access by the U.K. government—policies whose impact on and implications for research practice are in need of urgent evaluation, so as to decide on their eventual implementation elsewhere. This study is based on 22 in-depth interviews with U.K. researchers in systems biology, synthetic biology, and bioinformatics, which were conducted between September 2013 and February 2014. Through an analysis of the interview transcripts, we identify seven core themes that characterize researchers’ understanding of openness in science and nine factors that shape the practice of openness in research. Our findings highlight the implications that Open Science policies can have for research processes and outcomes and provide recommendations for enhancing their content, effectiveness, and implementation.

## Introduction

Over the past decade, the Open Science movement has gained momentum among publishers, funders, institutions, and, most notably, practicing scientists. Open Science has been broadly defined as the sharing of resources and ideas and places emphasis on making these publicly and freely available for future use. The Open Science movement is closely tied to both the “Open Data” and “Open Access” movements, which promote the sharing of data and publications, respectively (Laakso et al., 2011). It is also closely tied to open source models of intellectual property (Kelty, 2008), open governance (Tkacz, 2012), and the ethics of science (Peters, 2013). Though the Open Science movement is diverse, its constituents share the key assumption that promoting “openness” (Willinsky, 2005)—of multiple things, for multiple groups of people, and at multiple levels and geographies—will foster equality, widen participation, and increase productivity and innovation in science (Hey, Tansley, & Tolle, 2009; The Royal Society, 2012b). Here, we take the Open Science movement to broadly include the access to, dissemination of, and reuse of publications, data, materials, and methods.

In everyday research, Open Science takes many forms. It can involve researchers (1) putting their data into online databases such as GenBank and Figshare, or into journal repositories; (2) developing international standards for data formatting, curation, and quality, as promoted by institutions like the European Bioinformatics Institute (EBI) and the National Center for Biotechnology Information (NCBI); (3) publishing in open access journals like the Public Library of Science (PLoS), or publishing open access articles in journals like *Nature*, *Science*, and *Proceedings of the National Academy of Sciences*; or (4) creating software, models, or materials that can be of use across a variety of projects, labs, and disciplines, such as the BioBricks initiative in synthetic biology.

The Open Science movement has gained increased visibility and influence for a number of reasons. These range from scientific advances, such as recent developments in computing and communication technologies and the rise of “Big Data,” to political and economic factors, including the interest of European and North American governments in reinforcing the transparency and accountability of research processes, so as to renew public trust in science-based policies (Leonelli, 2013).

Despite widespread recognition of the value of Open Science and “openness” more broadly, proponents of Open Science differ on how they interpret the norm of “openness” in research, and on what they see as the best procedures to practice and encourage it. As other scholars have noted, there is little consensus over what is meant by, or how to practice, openness in science (Borgman, 2012; Edwards, Mayernik, Batcheller, Bowker, & Borgman, 2011; Grand, Wilkinson, Bultitude, & Winfield, 2016; Grubb & Easterbrook, 2011; Wallis & Borgman, 2011; Wallis, Rolando, & Borgman, 2013; Wynholds, Wallis, Borgman, Sands, & Traweek, 2012), and consequently little clarity around how the implementation and enforcement of Open Science should occur. Policies have different terms and requirements for researchers (Corrall & Pinfield, 2014), institutions have different infrastructures for repositories and databases, and scholarly communities have different commitments and goals. This often means that researchers do not know how, and in what way, to practice Open Science (Ferguson, 2014).

In this article, instead of defining “openness” in research a priori, we examine how researchers understand and enact “openness” in their everyday working lives. From an analysis of interviews carried out with scientific researchers in the United Kingdom—whose notions and practices of openness have in part been shaped by policies recently introduced by the U.K. government (Research Councils UK, 2013b; The Wellcome Trust, 2012)—we identify seven core themes that characterize the understanding and practice of openness in science, as well as nine factors that shape openness in practice. While doing so, we also acknowledge that openness is never singular or stable (Hilgartner & Brandt-Rauf, 1994; Kelty, 2008; Mauthner & Parry, 2013) and that openness in science varies with the context and contours of research.

By unravelling the significance of openness in everyday scientific work, we explore the practical implications of Open Science policies—whose implementation and institutionalization remains controversial and underinvestigated—for research processes and outcomes. We also highlight the challenges and opportunities facing the adoption and implementation of Open Science policies in the United Kingdom, thus providing insights that may inform future Open Science policies in the United Kingdom and elsewhere.

## The U.K. Open Science Landscape

Governments and funding agencies worldwide have begun to support the idea of “openness” as a crucial component of scientific research, particularly through the establishment of Open Science and Open Access policies and guidelines. Our study focuses on the United Kingdom as one locale—among many—in which openness is beginning to figure into the landscape of scientific research.^1^ In the following section, we provide a brief overview of the policy context in which notions of “openness” are developing and playing out within the United Kingdom and point to the unique timelines and occurrences of U.K. Open Science policies. This is instructive for the study and implementation of Open Science policies in other countries and internationally, since the United Kingdom, as we detail below, has played a pioneering role in introducing formal guidelines on this topic, and it therefore provides an excellent case study to examine the relation between such guidelines and existing research practices and understandings.

When we conducted this study in 2013, the U.K. government had recently established the RCUK Open Access Policy, which focuses on “unrestricted, on-line access to peer-reviewed and published research papers, free of any access charge” (Research Councils UK, 2013b, p. 1). The policy mandates that all publications from publically funded research in the United Kingdom be made available openly via two models of access: “gold,” in which authors (or their institutions) pay a fee to publishers to make their work freely available on publishers’ websites, or “green,” in which authors deposit peer-reviewed manuscripts in publicly accessible repositories (Björk et al., 2010). This policy followed from the recommendations of the 2012 government-commissioned Finch Report (Finch, 2012), which proposed to “minimize restrictions on the rights of use and re-use, especially for noncommercial purposes, and on the ability to use the attest tools and services to organize and manipulate text and other content.”

The U.K. government’s interpretation of Open Access, as expounded in these documents, is by no means the only existing approach to openness in research. Although proponents of Open Science have recognized that access to published research outputs may be the most straightforward and feasible recommendation in the short term, they have also emphasized the key role played by research data, biological materials, and methods. For example, The Royal Society’s seminal 2012 report *Science as an Open Enterprise* emphasizes the interrelatedness of research materials, data, and publications, and the subsequent need to consider Open Science more broadly (The Royal Society, 2012b). Formalized guidelines for Open Science, however, are absent from RCUK’s Policy on Open Access. Although the policy encourages the sharing of data, methods, and materials, it does not make sharing mandatory (Groves & Godlee, 2012), nor does it provide explicit suggestions for how sharing might occur and be regulated (Research Councils UK, 2013b, p. 4).^2^ The policy states:

As part of supporting the drive for openness and transparency in research, and to ensure that researchers think about data access issues, the policy requires all research papers, if applicable, to include a statement on how underlying research materials, such as data, samples or models, can be accessed. However, the policy does not require that the data must be made open. . . . If there are considered to be compelling reasons to protect access to the data. (Research Councils UK, 2013b, p. 4)

Such guidelines encourage but do not mandate the sharing of data, methods, and materials and do not provide explicit suggestions for how sharing might occur and be regulated (Digital Curation Centre, 2014). This is understandable given the variety of constraints and conditions relevant to the sharing of research materials other than publications, and yet it creates considerable confusion and disagreement among researchers, in their efforts to prioritize and strategize research outputs.

Recognizing the importance of access to data, materials, and methods, a number of governmental bodies, funders, journals, and communities have taken practical steps to outline broader Open Science guidance and policies that reach beyond open access to publications. For example, the League of European Research Universities issued a Roadmap for the handling and management of Open Data (LERU Research Data Working Group 2013) and Science International, an umbrella heading bringing together the World Academy of Science, the International Council of Science, the InterAcademy Partnership, and the International Council for Social Science, published a set of recommendations under the heading of *Open Data in a Big Data World* (Science International, 2015). RCUK, the Wellcome Trust, and the Royal Society have released statements encouraging grantees to publish data sets and methods alongside research findings (Research Councils UK, 2013a; The Royal Society, 2012a; The Wellcome Trust, 2013). Open access journals such as *eLife* and *PLoS* have established guidelines for depositing supplementary data. Recently, dedicated “data journals”—including *GigaScience, F1000Research*, and the *Nature* publication *Scientific Data*—and data repositories—including Dryad and Figshare—have also arisen. At the same time, scholarly communities have attempted to develop “altmetrics,” nontraditional metrics seeking to move beyond citation impact metrics such as impact factor and *h*-index, and which include metrics about data and software downloads, blog and website impact, and twitter (Piwowar, 2013).

Despite such statements and guidelines, the dissemination of data, biological materials, and methods is neither obligatory nor policed, creating a wide range of practices and notions of “openness.” As a result, researchers do not widely use repositories and databases, lack centralized standards and formats for circulating and ensuring the quality of their work, and are confused as to at what point in time and with what data they should be open (Nelson, 2009; Tenopir et al., 2015). Consequently, the United Kingdom’s current Open Science policy creates challenges at the levels of everyday research, institutional practice, and governmental policy.

## Method

This article is based on pilot project, which generated semistructured interviews with 22 principal investigators (PIs) aged approximately 35 to 60 years, who work in the fields of systems biology, synthetic biology, and bioinformatics, and who hold senior positions in 11 higher-education institutions in the United Kingdom. Interviewees were selected with snowball and convenience sampling techniques, based on the PIs’ ongoing working relationships with relevant scientists, and taking into account their prominence in their fields (as evidenced by their publications and public profiles), their experience in internationally recognized fields of research, their existing interest and involvement in Open Science discussions and practices, and their availability for the study (see Table 1).

**Table 1.** Interviewees by Research Field.

Institution	Department	Area of research
Imperial College London	Department of Medicine	Protein crystallography and synthetic biology
University of Aberdeen	School of Natural and Computing Sciences	Biochemical engineering of natural products
	Institute of Biological and Environmental Sciences	Environmental toxicity and bio-assays
University of Bath	Department of Biology & Biochemistry	Microbial metabolic engineering
University of Cambridge	Department of Plant Sciences	Plant synthetic biology and computational modeling
University College London	Department of Biochemical Engineering	Biochemical engineering of pharmaceuticals and biocatalysis
	Department of Biochemical Engineering	Biochemical engineering and synthetic biology of microorganisms
University of Edinburgh	MRC Institute of Genetics and Molecular Medicine	Network biology of cancer
	MRC Institute of Genetics and Molecular Medicine	Comparative genomics of model organism development
	School of Biological Sciences	Systems biology of plant circadian rhythms
University of Exeter	College of Life and Environmental Sciences	Plant cell signaling and bioenergy
University of Manchester	Faculty of Life Sciences	Cell signaling and imaging
	School of Computer Science	Computational and systems biology of metabolic signaling networks
	Faculty of Life Sciences	Small signaling molecules in microbes
	Faculty of Life Sciences	Computational biology for complex biological systems
University of Warwick	School of Life Sciences	Evolutionary systems biology and synthetic biology
	School of Engineering	Systems and control theory for synthetic biology
	Warwick Systems Biology Centre	Computational modelling and quantitative imaging of cell motion
University of York	Department of Biology	Biochemical engineering in plants
European Bioinformatics Institute (EBI)	NA	Chemoinformatics and metabolism
	NA	Bioinformatics of protein and RNA sequences
	NA	Population genomics and phenotyping

This research was supported by an ESRC Cross-Linking Grant between the Exeter Centre for the Study of the Life Sciences (Egenis) and the Edinburgh Institute for Innovation Generation in the Life Sciences (Innogen). The authors contributed as follows: Levin and Weckowska conducted the interviews; Leonelli and Levin analyzed the interview transcripts and drafted the article; Castle, Dupré, and Leonelli conceived of the study, and led its design and implementation.

The interviews lasted an average of 2 hours and took place between September 2013 and January 2014. The interview questions asked researchers about their understanding of “openness” in science and experiences with Open Access, Open Data, and Open Innovation, and aimed to explore researchers’ experiences and practices in relation to changing U.K. government policies. Our interviews sought to document scientists’ experiences, understandings, and practices of “openness,” based on questions about how, when, with whom, and why researchers shared or made available papers, data, biological materials, or methods. The interviews were semistructured, prompting researchers to explicitly articulate their conceptions of and reactions to “openness” in science. They were also open-ended, leaving interviewees free to form and express multiple, and sometimes contradictory, associations with this terminology.

The ensuing interview data were analyzed according to the major themes of broad notions and experiences of openness, experiences with open access, experiences with open data, and conditions and influences on the practice of openness. The themes were generated by a thematic analysis, in which the authors coded the interviews with the software program NVivo. The selected themes were included in the study if they were referred by at least three different interviewees. Notably, the ways in which themes were mentioned by interviewees varied widely, and each interviewee touched upon a different set of themes, making it impossible to establish overarching clusters of themes that recur together. Rather than showing the existence of well-defined and different “cultures of openness,” these interviews reflect a highly fragmented landscape, where each individual PI associated different issues with the notion and practice of openness, even within the same field. It is also important to note that no one theme dominated over others in terms of the number of researchers who mentioned it. The number of times that different themes were mentioned were fairly even over the whole sample, and thus do not provide insight into how such aspects are or should be ranked in relation to each other (an issue that should be addressed by a follow-up study).

The researchers selected for this study are not representative of the U.K. research community as a whole, but rather a representative sample of researchers who contribute to and/or have been affected by the U.K. Open Science Movement. Given the diversity of views encountered in this research, it should be made clear that not all interviewees shared the views and comments analyzed in this article. Given the size of our sample, we also stress that our findings do not aim to give an exhaustive or all-encompassing view of how scientific researchers understand and practice Open Science. We explicitly chose interviewees that had made public statements and/or performed at least part of their work in the spirit of Open Science (for instance, by setting up a public database or publicly committing to publish their papers in Open Access formats), so as to be able to harness existing understandings of the notion of “openness,” and investigate the relation between such understandings and the Open Science guidelines implemented by the U.K. government and funding bodies. This of course leaves out viewpoints of investigators who have an understanding of openness without being publicly involved with Open Science practices, thus potentially only exploring some of the existing diversity of perspectives. Furthermore, while our interviewees hailed from a variety of research specialties, sizes of laboratories and fields, and levels of collaboration with industrial partners, their views as PIs may not be representative of the views of other researchers within their laboratories, such as students, junior researchers, or technicians. Nevertheless, the interviews still capture a wide variety of perspectives. Some researchers were actively involved in Open Science through the development of community databases and infrastructures, or the establishment of standards and guidelines. Some encountered Open Science practices through increasingly interdisciplinary, collaborative, or computational work. Others engaged in a mixture of open and proprietary practices through their involvement with privately/industry-funded research. Thus, the interviews illustrate at least some of the diversity in the ideas and practices that characterize “openness”—or the lack thereof—in science, providing empirical grounds for future studies of other disciplines and national contexts.

## Biomedical Researchers’ Understandings of Openness

In this section, we analyze the range of meanings, experiences, and practices that researchers attribute to openness in science. When asked to reflect on the practices and ideologies that characterized and enabled openness in science, researchers emphasized the importance of thinking about openness in relation to all components of research, including data, models, software, papers, and materials such as experimental samples, plasmids, and animals. They equated openness with sharing, freedom, communication, and a communal norm.

Many researchers also acknowledged that openness was a “hot topic” or an “overhyped ideal,” and instead of defining it in terms of what it enabled and made possible, they chose to define it *in terms of what it is not*. Hence, openness was described as the opposite of “hiding,” “secrecy,” and “closing up.” Openness was also framed as a response to past periods of “closure,” in which there were broad-scale issues of access to community resources and ideas. Negative experiences with the commercial and closed nature of various types of research, for example, with the closed databases in structural chemistry, as well as restricted access to early versions of the Human Genome promoted by the Celera Corporation, were cited as reasons for pursuing a more open approach to science.

Overall, the accounts provided by researchers allowed us to identify seven core themes that characterize their understandings, experiences, and practices of openness in science.

### The Timely Donation of and Access to Research Components

Researchers highlighted the importance of submitting data, models, and other resources to established databases, and they emphasized the importance of facilitating access to resources through the creation and maintenance of fully open or managed-access databases (see Roche et al., 2014). Researchers also highlighted the importance of the manner—how and when—in which such donation and access occurred, placing value on the timely release of data, models, and biological materials (see Grand et al., 2016).

There is an open source principle [of] publish early, publish often. If you have some piece of working code, it doesn’t have to be complete, it just has to do something, and it has to work. . . . You [should] publish it in an open source repository, and then people can immediately work with it. (Researcher in chemoinformatics and metabolism)

Such timeliness, however, was balanced with a need to promote collaborations, protect attribution and credit, and to ensure quality and standards. For example, the donation of data to databases before publication remained controversial, due to concerns over being “scooped,” or of having their research ideas or findings published by another group first (see Grubb & Easterbrook, 2011). Researchers working with biochemical data also highlighted the importance of access to resources generated in the past, particularly in laboratories that contained collections of analog data (e.g., in paper files, images, or even Fortran punch cards), or in fields that relied on past literature that was only available via journal subscription.

### Standards for the Format and Quality of Research Components

Researchers emphasized that the existence of and adherence to various standards, which governed both the format and quality of data, enabled the use, reuse, and circulation of resources (see Neylon, 2012). This was important for making sure that work was not repeated, and for enabling the sharing and circulation of ideas and resources within research communities. However, researchers working in niche areas, where there were no standards, or where there was no consensus over which standard to use, struggled to reuse and disseminate research materials.

*With modelling it’s [often] that you get code that looks like spaghetti, right, and you can’t do anything with it. Again, you could say, “Okay, these guys are being open, they give you all the models they have,” but I can’t do anything with it.* . . . *[These] are things that people have already thought about, and now most journals will require you to submit models* . . . *in a certain format. People have developed standard languages for sharing models.* . . . *I think it boils down to the scientist to ensure that their things are reproducible, available, and understandable. If there are standards for what they do, they should use those standards. (Researcher in* evolutionary systems biology and synthetic biology)

For those involved in computational research, the donation of source code, instead of binary files, enabled researchers to access and modify software tools. For those involved in emerging fields of research, the use of standard data formats, such as the Systems Biology Markup Language, ensured that researchers would have access to high-quality data in online repositories.

### Metadata and Annotation

Researchers emphasized that the annotation of data, in particular the addition of metadata to large data sets, improved the quality and usefulness of resources (see Sansone et al., 2012). According to researchers, metadata provided the experimental conditions in which data were acquired and processed, or the internal logic with which data were analyzed. Many noted that metadata enabled data to become useful rather than simply available, and they provided additional information about how resources had been generated or used.

[Experimental data] is not useful for modelers unless it’s really carefully described. You have to have very good metadata in order to understand what the experiment was, so [you] can use it appropriately in models. (Researcher in systems biology of plant circadian rhythms)

The mandatory annotation of data on submission to repositories, however, remained controversial, due to concerns that it would require additional labor or interfere with the timeliness of donation.

### Collaboration and Cooperation With Peers and Communities

Some researchers associated openness with informal sharing of knowledge and research materials. Researchers emphasized that working with other academics or institutions facilitated the sharing of resources, labor, or ideas (see Evans, 2010). They highlighted the importance of informal sharing strategies, such as word-of-mouth, email, and postal exchanges, particularly where formal infrastructures were restrictive or absent.

We all know what [everyone else in the field] is doing. We just need an email to get anything that [the others] have. These informal networks are very important for distribution information to us. (Researcher in systems and control theory for synthetic biology)

For many, cooperation facilitated greater research output and productivity, as researchers mutually benefited from increased expertise and research capacity, and they formed ties that led to lasting collaborations. For others, cooperation provided tools and platforms to better the community of researchers, increasing the reproducibility of research, preventing the duplication of effort or loss of knowledge, and ultimately leading to more rigorous results and methods.

### Freedom to Choose Venues and Strategies for Disseminating Research Components

Researchers emphasized the importance of being able to choose the journals, databases, and repositories through which papers, data, and methods were disseminated, without being constrained by publishing costs or paywalls (see Gaule & Maystre, 2011). They highlighted that the choice of journals was never an easy task and involved balancing multiple considerations, such as author-processing charges, open access, quality and reputation, impact factor, and specialization (see Editors, 2006).

We are fortunate, we are one of the universities that got a big block grant by the research councils to publish the papers, and . . . I won’t need to pay the fees for it to be open access. . . . [So] at the moment, I’m not picking a journal by its price, I’m picking a journal by either what it publishes or . . . because it was a special issue that I really wanted to publish in: it was 100 years commemoration of a very famous paper, and it was going to be in that outlet. (Researcher in computational and systems biology of metabolic signaling networks)

Many researchers asserted that the existence of paywalls, or of non-user-friendly repositories, was detrimental to the access and dissemination of knowledge. Others worried that the central management of open access funds by universities, rather than via the budgets within grants, would constrain the freedom to publish, for example, if money were to run out or be poorly managed, or if such systems were to privilege those publishing early with a “first come, first serve” attitude. This latter concern is of course unique to scientific credit systems that operate by allocating chunks of funding to universities instead of researchers, and yet it is relevant more internationally as a warning against the problems that can arise when implementing such a mechanism.

### Transparent Peer Review Systems

Some researchers emphasized that openness should entail the transparency of peer review procedures as it increased the accountability and fairness of the publication process (see Ware, 2008). They saw anonymity as a barrier to the quality and honesty of referee reports, claiming that there was no mechanism to hold reviewers accountable for their comments or criticisms. Some suggested that “open” peer review should include access to the full range of data and materials analyzed within the article, in order to improve the quality of the published results through secondary and external validation.

Personally, I would favor a model which is not . . . based on anonymous peer review. Where actually your peers have to stand behind their decision, and clearly say why they think a paper should be accepted or not. That might eventually lead to a very different model for publishing science data, which could be radically different from the current one. (Researcher in computational modelling and quantitative imaging of cell motion)

This requirement for data and materials remained controversial, however, due to concerns that the already voluntary peer-review system would become increasingly cumbersome and slow. Some researchers highlighted the importance of radical new approaches to publishing, such as pre-print archives like arXiv, which were common in physics but not yet in biology (see Desjardins-Proulx et al., 2013).

### Access to Research Components in Non-Western and/or Nonacademic Contexts

Researchers highlighted a moral duty to provide access to data and publications in locales where there were fewer resources or infrastructures available, be they physical, economic, or intellectual (see Lezaun & Montgomery, 2015; Bezuidenout et al, in press). They emphasized the importance of “giving back” to society, and on making resources available to developing countries or less well-funded universities.

We should be trying to nurture conditions whereby data sharing is possible. That’s global conditions, so it’s a political question, [it’s a] current challenge. How do we foster these conditions whereby organizations and nations are in a position where they feel they can cooperate with one another? (Researcher in network biology of cancer)

Some researchers also placed value on making resources available to industry, saying that it was “fair,” and also a way to enhance the productivity of the economy. Enabling access to publications for industry, however, was a contentious topic, due to concerns that academic institutions that paid Open Access fees were enabling industries to derive potential profit from publically sponsored research.

## Factors Affecting the Practice of Openness in Science

In this section, we analyze the range of factors that shape researchers’ experiences and practices of openness, as they occur in varying contexts at the levels of everyday scientific work, institutional structures, and governmental policies. The focus of our analysis thus shifts from (1) the meanings and practices that define researchers’ understandings of openness in science to (2) the external factors that affect the practice of open science. In doing so, we reflect on how such factors have either negative or positive effects on the enactment of openness, depending on the contexts in which researchers work (Haeussler, 2011).

Overall, we identified nine factors that researchers thought to be crucial to openness in science.

### The Existence of Repositories and Databases for Data, Materials, Software, and Models

Researchers emphasized that repositories and databases, which were tied to the development of standards and metadata, affected their ability to access, reuse, and disseminate research materials. Most researchers emphasized the challenges associated with placing data within the supplementary information of journal articles, which limited the amount of data that could be deposited and often made it accessible in a way that was not user-friendly or particularly useful (see Fenner, 2010).

You can make a lot of data available to people and say “Here’s a big zip file, go get it.” But [people] can open up a zip file and get a lot of directories and get lots of files. So what? The researcher can say they’ve made it public, but it’s potentially of no use to anybody. (Researcher in comparative genomics of model organism development)

Many others highlighted the inadequacy of existing repositories and databases. For example, researchers involved in quantitative imaging emphasized the challenges involved in storing files that were very large, or which were generated in nonstandard formats, within public databases that had been developed with particular formats or standards in mind. Other researchers in emerging fields of research, such as computational modeling and metabolomics, stressed their difficulties in reusing and disseminating novel types or formats of data, for which there were no central databases or established standards.

### The Competitiveness of Academic Fields

Researchers emphasized that the competitiveness of a given field influenced their ability to collaborate and share research with peers (see Haeussler, 2011). Some researchers working in emerging and less competitive fields, for example, in some areas of systems and synthetic biology, found it easier to engage in open practices, which subsequently helped them create collaborations and promote the growth and visibility of their research (see Acord & Harley, 2013).

People generate these perceptions or views about things, that I’m sometimes not sure if they’re correct. . . . If you share your data too early you lose: I mean, that might be true for the very cutting-edge or very applied things. But most basic science is not really like that. I mean, for most of the stuff, there are only a handful of people who can follow that anyway. (Researcher in evolutionary systems biology and synthetic biology)

Other researchers working in highly competitive areas, for example, in biomedical research with animal models, felt pressured into withholding or selectively sharing resources due to fears of having their research ideas or publications stolen by other groups. Unsurprisingly, concerns over being “scooped” were more common for researchers engaged in human and animal work, which is more crowded and competitive, than for researchers engaged in the plant sciences.

### The Digital Nature of Research

Researchers emphasized that the data-intensive and computationally driven nature of their research made it such that sharing and dissemination were increasingly part of norms and institutionalized practices. Some researchers felt that digital objects were easier to share than physical materials or images, meaning that with the creation of digital objects they were more able to participate in open science initiatives. Other researchers, however, felt that the increasingly digital nature of research made it easier for others to steal data and ideas. Some researchers claimed that systems biology, because it was an interdisciplinary combination of mathematical, biological, and computer science expertise, was “ahead of the game” and had set the standard for open practices in other fields.

In my research area, computational systems biology, I think overall [we’re] probably more for openness than other areas. We are very much involved in standards, we were from the beginning . . . it’s all collaborative . . . we regularly do things as a consensus. . . . That makes is much more open because people are collaborating much more. (Researcher in computational and systems biology of metabolic signaling networks)

Researchers claimed that was due largely to the widespread availability of standards, data curation, and databases, which were integrated into the everyday research of systems biologists. Other researchers emphasized that in biological fields where computational methods were less established, there was a tendency to question the scientific usefulness and value of sharing and dissemination, and thus a lower rate of participation in open science initiatives.

### Credit Systems in Academic Research

Researchers emphasized that credit systems beyond the recognition of publications through metrics like impact factor or citation indices affected their ability to pursue curation, service, or infrastructural work. Although researchers acknowledged the existence of “Altmetrics,” nontraditional metrics for judging academic efforts and merit beyond journal publications (see Piwowar, 2013), they felt there was a tension between acting on behalf of the community and acting to further their own careers. Researchers struggled to engage in community-oriented work because of the time and effort required to format, curate, and make resources widely available (see Ankeny & Leonelli, 2015).

If your research is concerned with the development of cutting-edge evidence . . . you want to stay ahead of your competitors. Then you’re not keen on releasing that [research]. . . . It is a very, very hard problem, and one with a very high impact. . . . There is a big clash between trying to protect your own research, and then doing a community service and making it available for everyone. (Researcher in computational modelling and quantitative imaging of cell motion)

Researchers also expressed difficulties in establishing criteria for authorship and credit in collaborative projects, emphasizing that there was frequently an unclear division of labor among the researchers involved, as well as an unclear system for measuring or attributing labor.

### Career Structures in Academic Research

Researchers emphasized that career structures created pressures and expectations, which had varying effects on the ability of researchers at different stages of their careers to share or disseminate research. Researchers highlighted that PIs with established researchers had a high degree of flexibility with their outputs, as a strong track record of publications enabled them to disseminate research outputs or publications in ways that defied traditional credit systems like impact factor or citation indices. Researchers highlighted that, in contrast, younger scholars were more restricted in their ability to disseminate research outputs or publications, as they needed to cultivate a publication record in order to advance their career status.

[When] the analysis of the data could yield a Nature publication on which the career of a few PhD students is relying, in this case, I’m happy to keep these data closed for half a year, for a certain embargo period, so that these people can prepare analysis and write their high-level publications. (Researcher in chemoinformatics and metabolism)

In such cases, journal selection was carried out on the basis of impact factors, and sharing was restricted to avoid conflicts or competition with other researchers.

### Collaborations With Industrial Partners, as Well as Attempts at Commercialization

Researchers emphasized that ties to industry placed constraints on the sharing and dissemination of resources and research findings (see Evans, 2010). Many researchers working on projects funded predominantly by industrial partners asserted that data, materials, and other resources were rarely made available outside of the collaboration and instead remained the property of companies. They also asserted that the exchange of knowledge and resources in such collaborations was often unequal, as companies restricted openness and exchange with legal instruments such as nondisclosure agreements (see Walsh & Huang, 2014). Other researchers, however, highlighted that ties to industry provided novel and beneficial access to resources like proprietary data sets or biological materials. Some researchers acknowledged that industry ties had delayed the timing and affected the content of published research outputs (see Blumenthal, Campbell, Anderson, Causino, & Louis, 1997).

If it’s an [industrial] collaboration . . . you want to publish, but they want to check [the publications] out. And typically, they have a 30 to 60 day period in which they can decide whether they want to do something, but there are delaying tactics . . . it’s [also] to make sure that there’s nothing [in the publication] that could reveal what they are doing. If you revealed the secrets of their assay, which you’ve got in-house under a confidentiality agreement, they want to know that you’re not going to publish that. (Researcher in biochemical engineering of natural products)

Some researchers acknowledged that patents had delayed their publications by several months, while others acknowledged that the granting of patents had led to restrictions on the amount of information that could be contained in follow-up publications (see Grubb & Easterbrook, 2011).

### Models and Guidelines for Intellectual Property

Researchers highlighted that different open- and closed-source approaches to intellectual property affected the sharing and dissemination of ideas and resources. Some researchers claimed that open-source license like Creative Commons, LGPL, or GNU benefited the development of shared resources and technologies, while others emphasized that commercialization continued to rely on patenting and trade secrets. Most researchers emphasized, however, that material transfer agreements were a significant barrier to sharing and disseminating research materials, despite the fact that they were used to control access to resources by university technology transfer offices. Researchers also highlighted that the timing—the point at which intellectual property protection was established—affected the ability of researchers to share and disseminate ideas and resources.

I had this funny conversation once with a guy . . . [who] set up a company: they had a lot of proprietary ways of doing [things] . . . and design[ing] experiments . . . I went up to him and said, “Well this sounds really fascinating, the company must be doing really well now?” And he said, “No, actually the company flopped . . . we were just too early with these ideas, we couldn’t really communicate them, too many people didn’t understand, and we couldn’t share them because everything was under IP agreements” . . . I think it’s a U-curve: if you do too much protection too early, even in cutting-edge industrial applied things, [it] can actually become detrimental. The timing is very important.(Researcher in evolutionary systems biology and synthetic biology)

Some researchers emphasized that the early application of closed intellectual property regimes could restrict the development of new fields or technologies, noting that it was not worth the effort to commercialize software or patent inventions unless they were “dead easy to copy.” Others emphasized that the late application of intellectual property protection could cause researchers to lose market opportunities or be beaten by competitors.

### Governmental Views on the Status and Social Role Played by Universities

Researchers emphasized that the funding climate established by the U.K. government provided strong incentives for universities to develop industrial and transnational academic collaborations, shaping researchers’ decisions to collaborate, secure funding, or pursue the commercialization of research. Researchers asserted the increasingly international, cross-institutional, and impact-focused nature of research constrained their ability to share or disseminate resources, as it created an increasingly competitive academic system.

[The Research Councils] are desperate to come up with projects . . . that look like they are going to generate industries, create wealth, have impact and all of these kinds of things. . . . [But] the fundamental point is that we don’t understand how these systems work. That is the problem that has to be solved, before you can develop the industrial technology. . . . I am not skeptical about the commercialisation of research, if that is what people want to do, or if that is appropriate or useful. But what I am skeptical about is Research Councils putting pressure on academics to do commercialisable research, and only do that. (Researcher in systems and control theory for synthetic biology)

Researchers also emphasized the challenges with using the “open” infrastructures, such as repositories for data and publications, whose development had been shaped by U.K. government policies. Many researchers highlighted that there was a great deal of confusion over how these infrastructures should be implemented, resulting in a perceived gap between university-level policies and the experiences and needs of researchers and laboratories.

### The Existence of Various, and at Times Conflicting, Government Policies on Open Science

Researchers highlighted that Open Access and Open Data policies not only encouraged the sharing and dissemination of publications and data but also affected decisions to pursue particular intellectual property licenses defining the rights of use, reuse, and sharing. Many researchers emphasized that the policies put forward by different governmental bodies placed confusing and competing demands on their time and efforts.

Licensing is very complicated. . . . I think a lot of people just stick their head in the sand and just get on and reuse content if they think it’s probably okay, but actually, in many cases, I think the reuses are probably not okay. For example, people think that I’m reusing my open access paper, so I’m going to take that figure and use it in a Wikipedia article. Turns out it’s a creative commons CC-BY-NC [license], so [it’s] non-commercial. You can’t put non-commercial stuff in Wikipedia, but people think [because] it’s open access [it’s okay] . . . [So] I’m pleased there seems to be a push from the funding agencies to move to CC-BY licensing . . . so that content can be re-used and doesn’t have annoying strings attached. (Researcher in bioinformatics of protein and RNA sequences)

Some questioned how the Higher Education Funding Council for England’s (HEFCE) requirements for the Research Excellent Framework, which encouraged research “impact” through activities like commercialization, could be made congruent with the RCUK Policy on Open Access, which encouraged the free dissemination of research outputs. Moreover, some researchers emphasized the confusion surrounding the selection of open-source licenses for research outputs, highlighting that licenses stipulating noncommercial reuse were allowed by HEFCE and RCUK, but proved problematic for the inclusion of a publication’s content in public forums like Wikipedia, and also for reuse by industry.

## Discussion: Implications for Open Science Policy

In this article, we document the various ways in which biomedical researchers in the United Kingdom understand and enact openness in their everyday working lives. In doing so, we identify the following understandings of openness, as well as the factors that influence the practice of openness (see Table 2).

**Table 2.** Overview of Thematic Analysis.

Biomedical researchers’ understandings of openness	Factors affecting the practice of openness in science
1. The timely donation of and access to research components	1. The existence of repositories and databases for data, materials, software, and models
2. Standards for the format and quality of research components	2. The competitiveness of academic fields
3. Metadata and annotation	3. The digital nature of research
4. Collaboration and cooperation with peers and communities	4. Credit systems in academic research
5. Freedom to choose venues and strategies for disseminating research	5. Career structures in academic research
6. Transparent peer review systems	6. Collaborations with industrial partners, as well as attempts at commercialization
7. Access to research components in non-Western and/or nonacademic contexts	7. Models and guidelines for intellectual property
	8. Governmental views on the status and social role played by universities
	9. The existence of various, and at times conflicting, government policies on Open Science

Overall, these factors range from everyday technical issues to broader level institutional and policy issues, and provide both opportunities and challenges to the practice of openness by researchers. Notably, some of these factors are correlated, for instance, the competitiveness of a field can affect the credit system used within it. Unravelling the specific relations between these factors is not within the scope of our analysis and data, but it certainly would be important to explore in future work. What our findings do highlight is how, in order to understand the implications of Open Science policies, close attention must be paid to the variety of forms that openness can take in different stages and locale of research practice. Taking this as a starting point has the potential to enlighten discussions of Open Science—and openness in science—in a several ways.

First, our analysis illustrates how decisions about what to make open, and how and when, can vary widely depending on a number of factors: the ethos and hierarchical structure of the research field and community, the varying degrees of technical difficulty and labor involved in disseminating resources and results, the existence of useable infrastructures, and the degree of competitiveness and commercial stakes around the given research activity. Research methods, processes, settings, and goals are highly contextual, such that Open Science policies need to remain sensitive to the diversity of research contexts to which they might, or might not, apply. Indeed, our findings demonstrate that the circumstances under which it is appropriate, ethical, and scientifically fruitful to share resources and results vary widely, even within specific subfields of the life sciences, such as systems and synthetic biology.^3^ Openness is not always warranted or useful, and certainly not as a blanket policy applying indiscriminately to all stages of research across different fields.

Unfortunately, the diversity and contextual nature of openness is not always taken into account within broad Open Science policies and recommendations. Given this observation, we suggest that devising common Open Science guidelines and policies, which embrace all research practices in all location and at all times, may not be the best strategy for promoting Open Science. This is particularly the case for Open Data, given the heterogeneity of data formats, sizes, standards, and repositories. For example, adopting Open Data policies that are too stringent may have negative effects on scientific research, by forcing scientists to disclose results and resources in ways that they deem useless or inappropriate, or by requiring openness at a stage of research where it is more likely to hamper than encourage progress.

Second, our analysis illustrates how scientific institutions, funding bodies, learned societies, biomedical industries, and publishers play key and underexamined roles in promoting and encouraging particular forms of scientific openness. As many scholars and scholarly bodies have already noted, the implementation of Open Science requires substantial shifts in outlook for multiple stakeholders (Ankeny & Leonelli, 2015; Piwowar, 2013; RECODE, 2015), a phenomenon which the European Commission has referred to in the past as “Science 2.0” (European Commission, 2014). Amid significant changes in the ways that research is funded, circulated, and evaluated, our analysis suggests that for Open Science policies to succeed in their aims to foster more productive, democratic, and egalitarian research practices, it will be crucial for the diverse stakeholders in scientific research to *cooperate* toward a consistent and helpful framing and implementation of these policies, so as to avoid placing conflicting demands on the researchers involved; and indeed, that such cooperation needs to revolve around researchers’ own perspectives and experiences, so as to mediate between the wish for systemic change and the need to respect existing material and conceptual constraints, research demands, and ethical concerns.

While Open Science policies must remain responsive to diverse situations and contexts, they should also clearly assign responsibilities to each type of institution involved, again so as to ensure that researchers receive consistent and supportive advice on how to negotiate the various hurdles involved in sharing resources and ideas. This would help mitigate the confusion over the perceived conflicts between different policies and requirements for research across institutions, and the related confusion about what demands, evaluation criteria, and policies researchers should prioritize in their everyday work. As also recommended by the LERU Roadmap for Research Data (LERU Research Data Working Group, 2013) and the recent Science International Accord (Science International, 2015), funding bodies, learned societies, publishers, and universities should work together to help researchers evaluate and determine what should be disclosed, how, and at what point of the research process. For instance, learned societies could provide specialist assistance with the development of Data Management Plans or data infrastructures (Leonelli, Spichtinger, & Prainsack, 2015), funding bodies could create incentives for researchers to consult each other over the advantages and disadvantages of sharing specific elements of their work, universities could take more responsibility in helping researchers to examine the ethical accountabilities involved in each proposed project (Nuffield Council on Bioethics, 2015), and publishers could facilitate and reward participation in peer review groups aimed specifically at evaluating outputs other than journal publications.

Third, our findings show that meanings and practices associated with openness in science have a more complex and extensive history than what is currently considered by funding agencies and government bodies, and that this matters when attempting to assess which policies may best fit any given field of inquiry. Funding bodies should pay more attention to the collection and dissemination of data produced in the past, so as to better exploit previous investment, and to avoid creating a “presentist” culture of research, where the only outputs that matter for knowledge production are those produced now or in the future (see also Leonelli, 2016). This would also increase awareness of which resources are worth preserving, and which resources are too difficult and costly to store and disseminate efficiently and fruitfully.

Fourth, this analysis points to policy recommendations concerning the ways in which scientific reporting, funding, and credit systems should be managed to foster Open Science. In particular, the challenges identified in relation to commercialization and collaborations with industry support a recommendation that Open Science (and specifically Open Data) policies include clear instructions for researchers involved in commercial partnerships or commercialization efforts. The concerns identified in relation to competitiveness and career structures point to the need for institutions to reform their promotion and tenure policies, so as to support Open Science. And since administrative burden was identified as a challenge in relation to many of the themes under discussion, greater investment should be placed toward supporting the infrastructures and personnel that can help to implement Open Science policies, and particularly data management and curation services.

Finally, this study demonstrates that there is a need for more empirical research showing how Open Science policies, including Open Access and Open Data policies, have implications for peer review procedures, credit and excellence measures, and the sharing (or not) of research materials. While our exploratory study is a step in this direction, work remains to be done examining the other disciplines, locations, and/or timescales in which Open Science occurs, and also comparing the national contexts in which science policy and practices are embedded. This type of social science research can help capture and realize the chief aims of the Open Science movement, by identifying diverse and contextual ways to increase the excellence, reliability, accountability, and transparency of research. This is particularly relevant given that open science policies are a moving target, with new guidelines and mandates being released and amended on a regular basis, which creates a unique opportunity for empirical findings such as those discussed to directly inform the evolving policy landscape.

## Notes

1. While the United Kingdom has been a pioneering site for the development of Open Science policy, there are other places throughout the world experiencing fast developments and debates around Open Science. For example, open access has been a key topic in recent public policy debates in the United States, after the U.S. government launched an open access policy in 2013, extending its existing open access policy—established in 2008 and covering only biomedical research—to include all publicly funded research; and the European Commission launched an Open Science initiative in 2016, aiming to make all European Union–funded research “open.”

2. Officials within RCUK and the individual research councils are keenly aware of this policy gap and are actively researching incentives for data sharing, for example, through consultations such as the one carried out by the Higher Education Funding Council for England in the wake of the 2014 Research Excellent Framework (HEFCE, 2014).

3. It may be argued that these fields are particularly diverse, given their recent emergence.
